# Repurposing of Tibolone in Alzheimer’s Disease

**DOI:** 10.3390/biom13071115

**Published:** 2023-07-13

**Authors:** George E. Barreto

**Affiliations:** Department of Biological Sciences, University of Limerick, V94 T9PX Limerick, Ireland; george.barreto@ul.ie

**Keywords:** Alzheimer’s disease, tibolone, mitochondria, neuroglobin, oestrogen receptors

## Abstract

Alzheimer’s disease (AD) is a debilitating neurodegenerative disease characterised by the accumulation of amyloid-beta and tau in the brain, leading to the progressive loss of memory and cognition. The causes of its pathogenesis are still not fully understood, but some risk factors, such as age, genetics, and hormones, may play a crucial role. Studies show that postmenopausal women have a higher risk of developing AD, possibly due to the decrease in hormone levels, especially oestrogen, which may be directly related to a reduction in the activity of oestrogen receptors, especially beta (ERβ), which favours a more hostile cellular environment, leading to mitochondrial dysfunction, mainly affecting key processes related to transport, metabolism, and oxidative phosphorylation. Given the influence of hormones on biological processes at the mitochondrial level, hormone therapies are of clinical interest to reduce the risk or delay the onset of symptoms associated with AD. One drug with such potential is tibolone, which is used in clinics to treat menopause-related symptoms. It can reduce amyloid burden and have benefits on mitochondrial integrity and dynamics. Many of its protective effects are mediated through steroid receptors and may also be related to neuroglobin, whose elevated levels have been shown to protect against neurological diseases. Its importance has increased exponentially due to its implication in the pathogenesis of AD. In this review, we discuss recent advances in tibolone, focusing on its mitochondrial-protective effects, and highlight how valuable this compound could be as a therapeutic alternative to mitigate the molecular pathways characteristic of AD.

## 1. Introduction

Alzheimer’s disease (AD) is a neurodegenerative disease characterised by the accumulation of amyloid-beta (Aβ) and tau in the brain, which can lead to severe cognitive impairment and memory loss. Recent studies show that the incidence and prevalence of AD have increased exponentially worldwide in recent years, and the trend is similar in Europe, where, according to a published study, ~16.3 million people (3.28% of the population) will be living with dementia by 2050 [1], with AD being the most common form of dementia. This finding suggests that with the aging of the population, it is critical and urgent to implement public policies aimed at improving prevention, diagnosis, and more specific therapies applied in the earlier stages of the disease to mitigate the exponential growth of cases [2,3,4,5].

Although the exact causes of the pathogenesis of AD are still not fully understood, some risk factors have been sought, including age, genetics, lifestyle, and hormones [6,7]. Regarding the last factor, some studies have shown that women who go through menopause at an earlier age have a higher risk of developing AD [8]. Other studies also suggest that women who are menopausal before the age of 45 are more likely to develop the disease than women who go through menopause after the age of 45 [9,10,11]. These data suggest that a decline in hormone levels during the menopausal years may significantly increase the risk of developing AD [12,13], probably due to an accumulation of tau mainly in the entorhinal cortex [14], one of the first brain areas to be affected. In addition, the decline in circulating oestradiol has been shown to reduce oestrogen receptor beta (ERβ) transcriptional activity [15,16,17], which may promote a more pro-inflammatory environment [18], mitochondrial damage, and metabolic dysfunction [19,20,21] and be associated with the increased accumulation of Aβ [22]. Furthermore, single-nucleotide polymorphisms (SNPs) of the ERβ-encoding gene (*ESR2*) have also been associated with cognitive decline and an increased risk of AD in women [23,24,25,26], demonstrating the involvement of this receptor in the pathological mechanisms of the disease.

The mitochondrion is an organelle that is particularly affected by AD. It is still not well understood whether early molecular changes in this cellular compartment are the cause or consequence of the first signs of the pathology and when its dysfunction begins during the lifespan. Regardless, several mitochondrial proteins have been implicated, many of which are related to apoptosis, cell metabolism, and transport across mitochondrial membranes. Previous studies have reported that the voltage-dependent anion channel (VDAC), translocase of the outer mitochondrial membrane (TOM), and ATP synthase are all dysregulated in mouse models of AD and in the brains of AD patients [27,28,29,30,31]. These proteins may be regulated by estrogenic mechanisms, as the presence of ERβ [32], which interacts with mitochondrial respiratory complexes [33], suggests the modulation of all these metabolic and oxidative processes in the mitochondria. Interestingly, one of the proteins regulated by ERβ is neuroglobin (Ngb), a globin whose functions range from anti-apoptotic and anti-inflammatory processes to the regulation of mitochondrial metabolism [34,35,36]. The impairment of these proteins has a huge impact on the progression and development of AD, leading us to hypothesise that therapeutic strategies need to be more specifically targeted at preserving their functionality for better mitochondrial homeostasis.

Given the influence of hormones in controlling biological processes within the mitochondria, hormone replacement therapies have gained clinical importance in reducing the likelihood or delaying the onset of symptoms associated with AD. Our group has long been investigating the neuroprotective potential of tibolone, a pro-synthetic drug approved by the Food and Drug Administration (FDA) and used by women to reduce adverse menopausal symptoms, which has been shown to have broad anti-inflammatory and antioxidant effects [37] by activating oestrogen receptor alpha (ERα) and ERβ, progesterone (PGR) and androgen (AR) receptors [38,39]. It is still unclear whether women who take this compound have a lower risk of developing AD, or whether it delays the onset of the disease or relieves symptoms. Hormone therapy with oestrogens and progestins that are started earlier after menopause is associated with a lower risk of AD [8], in contrast to treatment started long after menopause, which may lead to a higher risk and worse outcome [40,41,42,43]. Rather than focusing on the benefits of this therapy as an adjuvant treatment in AD (see a previous review [44]), in this review, we will discuss how tibolone may be of clinical interest as an alternative therapy to combat the multiple mitochondria-related inflammatory and oxidative cascades that are hallmarks of AD.

## 2. Mitochondrial Dysfunction in Alzheimer’s Pathology

Cellular and molecular processes involving mitochondria in AD pathology have been extensively reported in recent years [45,46,47,48]. Despite the extensive literature on this topic, in the following sections, more focus is placed on how mitochondrial processes related to mitochondrial transport, apoptosis, and oxidative phosphorylation for the maintenance of redox homeostasis may be regulated by steroid receptors through tibolone and how the underlying mechanisms are most affected in AD.

### 2.1. Changes in Mitochondrial Transport by TOM Proteins

TOM proteins are particularly implicated in the pathology of AD. The TOM complex consists of several proteins (subunits), each with a specific function in the mitochondrial transport mechanism. With a total of seven subunits, TOM6, TOM5, TOM7, TOM22, TOM20, TOM40, and TOM70 are located in the outer mitochondrial membrane (OMM) [49], with TOM22 and TOM20 acting as proteins that recognise the N-terminal mitochondrial targeting sequence (MTS) of proteins whose final destination is the mitochondrion. On the other hand, TOM40 is one of the central proteins of this complex, forming a central channel through which these proteins with the MTS pass to be internalised by this organelle [50]. The other proteins, TOM5 and TOM6, as well as TOM7, are small compared to the others, but they help to stabilise this complex and regulate its activity. Because of its importance in transporting proteins that are critical for maintaining mitochondrial function, defects in this complex have been linked to AD. One of the studies demonstrating this association shows that the Aβ peptide uses this transporter to enter the mitochondria and localises to mitochondrial cristae [51]. This evidence leads to two possible explanations: (1) the formation of Aβ is not mitochondria-specific but (2) occurs by a mechanism not yet fully understood, possibly related to the fact that Aβ can cause mitochondrial membrane depolarisation, leading to greater internalisation of the peptide [52]. Another possible mechanism is related to the disruption of membranes, including mitochondrial membranes, that may have been caused by Aβ, which in turn facilitates its internalization.

In addition to the Aβ transport mechanism, one of the TOM complex proteins, TOM70 (also known as TOMM70), appears to play a key role in the pathology. Previous studies suggest that this protein is associated with the recognition of precursor proteins and is, therefore, a critical element of mitochondrial transport with regulatory capacity [53]. This protein is significantly reduced in the blood [27,53,54] and the brain [28] of AD patients, and a significant reduction in TOM70 was later confirmed in the hippocampus of APP/PS1 mice at 6 and 12 months of age [27]. This drastic reduction in mitochondrial transport mechanisms has serious implications not only for ATP generation and how this organelle mediates lipid and carbohydrate metabolism processes but also for more complex processes, such as neuronal development, calcium (Ca^2+^) signalling, and synaptic neurotransmission, which are classic hallmarks of AD.

### 2.2. Impairment of Mitochondrial Porins in AD

One of the best-known mitochondrial porins, the voltage-dependent anion channel (VDAC), is a transmembrane protein located in the OMM. Due to its strategic location, it is involved not only in almost all cellular processes but also in those related to apoptosis, metabolism, and mitochondrial dynamics [55].

Structurally, VDAC is a beta-barrel protein with 19 anti-parallel beta strands, each of which is a series of loops that will form the pore in the OMM [56]. VDAC has two subunits, VDAC1 and VDAC2 [56]. With a function similar to the TOM complex, VDAC also serves as a channel for the passage of molecules, but a requirement is its high permeability to small molecules, such as ATP, NADH, ADP, and metabolic substrates such as pyruvate, allowing an exchange between the cytosol and mitochondria for rapid processing [57]. In addition, VDAC also allows the passage of small ions such as Ca^2+^ and potassium (K^+^), which are essential ions for maintaining the mitochondrial membrane potential (ΔΨm) [57,58,59], thereby regulating mitochondrial redox homeostasis.

In addition to its critical role in mitochondrial trafficking, VDAC1 interacts with B-cell lymphoma 2 (Bcl-2) and may also be involved in the regulation of apoptosis [60]. Other proteins, such as the pro-apoptotic protein tBid, have been reported to co-regulate the VDAC channel [61], specifically subunit 2 (VDAC2) [62], suggesting the intrinsic regulation of the mitochondrial transition pore (mPTP), which is one of the first steps in the activation of apoptosis. It is thought that this regulatory mechanism of mitochondrial integrity may be altered in pathologies such as AD. Since both Aβ and hyperphosphorylated tau are the main components involved in the pathology, one of the first questions that remain unanswered is whether they interact directly or indirectly with some subunits of VDAC. A study by Manczag and Reddy in 2012 suggested that both Aβ and tau interact with subunit 1 (VDAC1), not only in APP, APP/PS1, and 3XTg.AD transgenic animals but also in the cortex of the brains of AD patients [30]. Two years later, in 2014, Fernandez-Echevarria et al. confirmed the previous results showing that VDAC1 is associated with lipid rafts of neurons in AD patients, in addition to reporting that amyloid precursor protein (APP) interacts with VDAC1 in the frontal and entorhinal cortices of AD patients at earlier stages of the pathology [63]. In addition to a direct interaction with VDAC1 [30], some post-translational modifications (PTMs) appear to be involved in AD. For example, Aβ induces VDAC1 dephosphorylation, and this process appears to occur in lipid rafts in AD brains [63]. The formation of this complex significantly alters the function of mitochondrial pores, with the direct consequence of mitochondrial dysfunction and ATP impairment, but it has not been investigated whether this is one of the pathophysiological mechanisms of AD in more advanced stages, or whether it could be used as a possible early marker of pathology. Further studies are needed to test this hypothesis.

### 2.3. Impaired Mitochondrial Respiration in AD via ATP Synthase

Dysfunction of mitochondrial transport processes by TOM and VDAC is expected to cause severe impairment of mitochondrial respiration and oxidative phosphorylation. Of particular interest because of its involvement in both processes, ATP synthase has also been implicated in AD. ATP synthase, also known as F1Fo-ATP synthase or complex V, is a highly conserved complex located in the inner mitochondrial membrane (IMM) and is responsible for the synthesis of ATP through the proton motive force generated by the flow of electrons pumped along the electron transport chain.

ATP synthase has two major subunits, F1 and Fo [64,65], with F1 composed of five other subunits (α, β, γ, δ, and ε) organised in a hexameric ring. The rotation of the F1-γ subunit, critical for ATP production, is driven by the proton gradient formed between the intermembrane space and the mitochondrial matrix, which induces conformational changes in the other subunits and thus generates sufficient energy for the phosphorylation of ADP to ATP [65,66]. On the other hand, Fo has three subunits (a, b, and c) that together form the proton channel that crosses the entire IMM [67]. This process is called oxidative phosphorylation and is the primary mechanism for the generation of intracellular ATP. In contrast to this process, ATP synthase can also work in the reverse mode, i.e., to hydrolyse ATP as one of the mechanisms to maintain ΔΨm, which is formed by the difference in the proton (H^+^) gradient between the mitochondrial matrix and the intermembrane space. ΔΨm can be considered an excellent indicator of mitochondrial function, as a reduction in its threshold is directly associated with several neuropathological processes, including AD. In this context, the activity of ATP synthase is significantly altered, leading directly to a decrease in ATP production and mitochondrial dysfunction [29,68]. Indeed, not only is the nuclear-encoded mRNA for some subunits of ATP synthase reduced in the cortex of AD patients [31], but Aβ also forms aggregates with the α-subunit of ATP synthase [69], suggesting a possible physical interaction that alters the ability of this complex to generate ATP. Dysregulation of the β-subunit of ATP synthase has also been reported [70]. As this complex is essential for the phosphorylation of ADP to ATP and the reduction of oxygen to produce H_2_O as the final part of oxidative phosphorylation, these molecular changes lead to an increase in oxidative stress processes and mitochondrial impairment. In the following section, we will discuss how to protect mitochondria from this oxidative damage using tibolone as a possible pharmacological approach.

## 3. Molecular Signature of Tibolone’s Actions in the Brain

Tibolone is a synthetic drug with predominantly tissue-dependent activity and can have estrogenic, androgenic, or progestogenic effects. It has been used in the clinic as a hormone therapy to relieve menopausal symptoms. Although tibolone improves bone density [71], increases sexual function and libido, and reduces hot flushes in women [72,73], many of the mechanisms by which tibolone exerts these effects are not well understood. This is largely due to its differential metabolism in distinct tissues and the lack of knowledge of its molecular mechanisms, which makes it very complex to study.

Tibolone is initially metabolised in the liver by several enzymes, including cytochromes P450 (CYP), UDP-glucuronosyltransferases (UGTs), and sulphotransferases. During metabolism, tibolone is converted to three metabolites, 3α-hydroxytibolone and 3β-hydroxytibolone, which have estrogenic activity by activating ERα and ERβ, and the Δ4-isomer metabolite formed by the reduction of the double bond in the A-ring of tibolone [39,74]. This metabolite is more likely to act through the activation of the AR and PGR. Interestingly, the metabolism of tibolone may be influenced by several factors, including age, sex, and pharmacological co-interaction with other drugs. Due to its multiple pharmacological actions, in the next sections, we will discuss how tibolone may have clinical utility as a therapy targeting mitochondria-dependent molecular pathways affected by AD.

### 3.1. Neuroglobin Upregulation as a Protective Mechanism

Investigating each pharmacological potential of tibolone in isolation is a difficult task due to its broad spectrum of action through steroid receptors and multiple cross-interactions with pathways related to apoptosis and mitochondrial dynamics. To screen for proteins that may influence many of these tibolone-induced molecular pathways, Avila Rodriguez et al. in 2014 showed that 10 nM tibolone upregulated neuroglobin (Ngb) in both T98G cells and primary mouse astrocytes subjected to metabolic dysfunction [21]. Ngb is a 151 aa protein belonging to the globin family. After its discovery in 2000, it was thought to have a function solely related to oxygen sensing and transport under hypoxic conditions, but it was later discovered that this protein is downstream of hypoxia-inducible factor 1 subunit alpha (HIF-1α) [75,76], a transcription factor that is activated under conditions of low cellular oxygen and plays a critical role in apoptosis by interacting with and stabilising p53 [77]. Upon stimulation, Ngb changes from a hexameric to a pentameric structure, increasing its peroxidase capacity and thus its ability to scavenge reactive oxygen (ROS) and nitrogen species (RNS). It has also been observed to bind to mitochondrial complex I and III proteins, where most ROS production occurs, making Ngb essential in the regulation of oxidative stress and energy metabolism [78]. Work from our group has also shown that Ngb-depleted cells (Ngb^−/−^) lose mitochondrial membrane potential (ΔΨm) along with increased levels of superoxide (O_2_^−^) and hydrogen peroxide (H_2_O_2_) while attenuating catalase and superoxide dismutase 2 (SOD_2_, mitochondrial), two antioxidant enzymes [79,80], suggesting a role for neuroglobin in regulating endogenous protective mechanisms that may be mitochondrial-dependent. In addition, the inhibition of ubiquinone oxidation at the Qi site of complex III by antimycin A, together with Ngb^−/−^, has a completely negative effect on ΔΨm and the survival proteins pAKT and pERK1/[79,80].

While Ngb attenuates ROS/NOS generation, it is likely that this protein also negatively regulates apoptosis [34,35,36,81,82]. The interaction of neuroglobin with cytochrome c disrupts caspase activation [83] and upregulates B-cell lymphoma 2 (Bcl-2) while downregulating caspase 3 and Bcl-2-associated X (Bax) in neurons [83]. Similarly, Ngb also exerts cell survival properties by interacting with G protein subunit alpha O1 (GNAO1); REL proto-oncogene, NF-KB subunit; and contactin-associated protein 2 (CNTNAP2), a neurexin family protein enriched at synapses involved in axonal growth [84]. Taken together, these studies suggest that crosstalk between membrane receptors and inflammatory pathways may be an important function of neuroglobin as an endogenous protective mechanism.

The neuroprotective mechanisms of Ngb may be mediated in part by ERβ [21] and appear to be mitochondria-specific. Previously, T98G cells were pre-treated with 10 nM tibolone or 10 nM 2,3-bis(4-hydroxyphenyl)propionitrile (DPN, an ERβ agonist) and then subjected to metabolic challenge for 24 h. Both cells treated with tibolone alone and those treated with the ERβ agonist showed similar results, with both compounds reducing the percentage of fragmented nuclei stained with Hoechst 33258, a sign of early apoptosis, and protecting mitochondria by preserving mitochondrial membrane potential, as measured by TMRM, and superoxide (O_2_^−^) generation, and finally by positively regulating Ngb. In contrast, the administration of phenyl-trifluoromethyl-pyrimidine phenol (PHTPP, an ERβ antagonist) 4 h prior to the addition of tibolone or DPN to cell cultures almost completely reversed the neuroprotective effect of tibolone and DPN, mainly by decreasing neuroglobin protein levels in both T98G cells and primary astrocyte cultures [21]. Similar effects have been observed in murine BV-2 microglia and T98G cells when subjected to palmitic acid lipotoxicity [19,20]. These previous findings are of interest for tibolone as a potential therapy for AD, as neuroglobin may have an intrinsic interaction with Aβ [85], and ERβ, if reduced in expression and, consequently, activity with the hormonal decline in women, may cease to exert its modulating effects on neuroglobin.

### 3.2. Regulation of Apoptosis, VDAC, and Mitochondrial Trafficking

Over the years, studies have expanded the role of Ngb, showing that its function is not limited to this and that it also has extensive anti-apoptotic and anti-inflammatory effects [34,35,36,81], making it interesting from a pharmacotherapeutic point of view.

Neuroglobin can interact with several proteins in the cytosol and at the mitochondrial level. As discussed before, under stress conditions such as inflammation and hypoxia, neuroglobin is located in the mitochondrion [79], where it interacts with mitochondrial complex I and III proteins and binds to cytochrome c, preventing its translocation to the cytosol and inhibiting apoptotic processes. As this protein is partly regulated by ERβ and appears to physically interact with VDAC [86], it may favour the regulation of the opening of the mitochondrial transition pore, thus preventing the exit of cytochrome c into the cytosol, in addition to being actively involved in calcium signalling via IP3Rs [87], and has been shown to be associated with ATP production [88]. As these mitochondrial processes are altered with age and in neurodegenerative pathologies, it has been suggested that neuroglobin is likely to be affected [85], particularly as one of its key endogenous regulators, ERβ, is also reduced in AD [89]. As hormonal fluctuation is a major risk factor for AD, it is unclear whether neuroglobin is altered during the female endocrine transition. Further studies are needed to confirm and validate neuroglobin expression in the female brain at different ages, especially during the perimenopausal transition to menopause.

In addition to upregulating neuroglobin, tibolone plays a critical regulatory role in apoptosis. A randomised clinical trial measured apoptotic markers in the blood of menopausal women using tibolone. The authors showed that postmenopausal women treated with tibolone for 6 months had reduced serum levels of sFasl (soluble Fas ligand, belonging to the TNF family) at 6 months (40.45 pg/mL) compared with baseline levels (53.8 pg/mL) [90]. Similarly, 2.5 mg of tibolone for 3 months was also able to significantly reduce Fas ligand levels in healthy postmenopausal women [91]. Previous studies from our group also show that 10 nM tibolone reduced levels of interleukin-6 (IL-6), which has been implicated in cell survival and apoptosis [92], in T98G cells exposed in vitro to 2 mM palmitic acid [93], a finding later confirmed by a network pharmacology study showing that IL-6 is one of the major druggable targets of tibolone [94]. In addition to reducing pro-inflammatory molecules such as IL-6, tibolone can also downregulate others, such as interleukin 1-B (IL-1B) and Toll-like receptor 4 (TLR4), and reduce the expression of Bcl-2-associated X (Bax), one of the central proteins in the apoptotic mechanism, after palmitic acid insult [19]. In an in vivo model of multiple sclerosis, animals injected with 0.08 mg kg^−1^ of tibolone showed less reactive gliosis, in parallel with a significant decrease in the overexpression of TLR4, high mobility group box 1 (HMGB1), NF-kB, and the NLR family pyrin domain–containing 3 (NLRP3) inflammasome, suggesting that tibolone may exert anti-apoptotic effects by reducing inflammation, possibly mediated by ERβ [19,20,21].

Whether tibolone modulates mitochondrial transport processes such as the TOM complex, either by physically binding to it or by regulating its expression, is not well understood. One of the few studies investigating the estrogenic regulation of mitochondrial translocases was performed by Schubert et al. in 2016 [95]. They did not focus exclusively on TOM, but on a protein that is part of the translocase located in the inner mitochondrial membrane, TIM23. In this study, female C57BL/6N mice were ovariectomised and pre-treated with a single injection of oestradiol (E2, 0.2 mg/kg) or ERβ agonist A (1.6 mg/kg) 24 h prior to cardiac ischaemia. Animals treated with oestradiol or the ERβ agonist showed reduced levels of apoptotic nuclei, cleaved caspase-9, and cytochrome c. In addition, the levels of both TIM23 and NADH:ubiquinone oxidoreductase complex assembly factor 8 (NDUF8) [95], a protein belonging to complex I of the electron transport chain, were significantly higher than in untreated animals, suggesting the involvement of ERβ in mediating the protective effects on mitochondrial integrity and metabolism. Further studies are needed to explore this issue further.

To further investigate how tibolone regulates mitochondrial protection mechanisms, human astrocytes treated with 10 nM tibolone were exposed to high concentrations of palmitic acid (2 mM), and the RNA was isolated, purified, and sent for sequencing. One of the most significant results is that a protein highly regulated by palmitic acid and tibolone was importin-7 (Ipo7 or Imp7) [96]. Among its functions, Imp7 is linked to the import of cellular components into the nucleus, where, together with the nucleoporin Nup358, it transports human telomerase (hTERT) [97], whose main function is the maintenance of telomeres [98] and which is composed of a catalytic subunit (TERT) and an RNA subunit (TERC). Interestingly, the catalytic subunit appears to have non-canonical functions (i.e., mitochondrial protection) other than those it normally regulates, i.e., telomeres, by modulating neuroprotective processes [93,99], possibly through nuclear factor kappa-light-chain-enhancer of activated B cells (NF-kB) [100]. In line with previous results, mitochondria isolated from HeLa cells and fluorescently labelled mtDNA were used to study mtDNA transport across the cell. After stimulating these cells with Imp7 and ran to activate the nuclear import mechanism, the presence of mtDNA in the nucleus was observed [101], demonstrating a crosstalk between mitochondrial material and regulatory processes at the nuclear level. Of particular interest in apoptosis, Imp7 inhibition attenuates ribosome biogenesis and activates cell cycle arrest dependent on p53 [102], a protein with a strong pro-apoptotic component, and certain post-translational modifications of this protein may be directly related to AD pathology [103].

## 4. Regulation of Aβ and Tau Hyperphosphorylation

Not surprisingly, both Aβ and tau significantly affect mitochondrial respiration and oxidative phosphorylation [104,105,106]. Perhaps one of the biggest unanswered questions is whether tibolone can prevent or reverse Aβ-induced mitochondrial dysfunction in AD, and how this would affect disease progression and cognitive dysfunction.

One of the first studies to demonstrate some benefits of tibolone on molecular pathways involved in AD pathogenesis came from Guerra-Araiza’s group in 2012. In this study, adult ovariectomised female rats injected with 0.5 mg/kg/day of tibolone for 2 months were tested for total tau, hyperphosphorylated tau (PHF-1), glycogen synthase kinase 3 beta (GSK3B), one of the kinases that can phosphorylate tau [107], and phosphorylated GSK3B, its inactive form. Treatment with tibolone significantly reduced the levels of hyperphosphorylated tau while increasing phosphorylated GSK3B in the hippocampus and cerebellum of ovariectomised animals. Later, using a mouse model of AD (3xTgAD), Segura-Uribe et al. (2022) confirmed the previous findings by showing that tibolone at 1 mg/kg reduced Aβ and tau in the hippocampus of transgenic animals, which was associated with a significant improvement in memory processes [108]. What would be the most immediate consequence of this in terms of cognitive function, and could it be said that there is an improvement in learning and memory processes at the hippocampal level? This is a complex question, but the fact that tibolone increased the inactive (phosphorylated) form of GSK3B may indicate the regulation of the endogenous mechanisms for tau phosphorylation.

Not only does GSK3B phosphorylate tau, but others, such as cyclin-dependent kinase 5 (cdk5), have also been reported [109,110]. In a previous study, tibolone increased cdk5 levels at low doses (0.01 mg/kg) but decreased them at higher doses (1.0 mg/kg) [111], suggesting a concentration-dependent effect. Separately, these effects of tibolone in old male animals are sufficient to improve neuronal function associated with learning and memory processes [111]. In further support of tibolone’s effects on synaptic processes, De Aguiar et al. (2006) showed that tibolone improves short-term and long-term memory in young, adult, and aged animals [111] and also controls anxiety behaviour in ovariectomised rats [112], possibly by protecting the cholinergic neurotransmission mechanism in the hippocampus [113]. In this regard, tibolone at 1 mg/kg/day given to male rats for 60 days increased acetylcholine (Ach) levels in the hippocampus. Cholinergic neurotransmission is essential for maintaining learning and memory processes [114], and the enzyme acetylcholinesterase (AChE) is altered in the brains of AD patients [114,115], reducing the pool of available Ach, which is associated with severe cognitive deficits [116,117,118]. As the metabolites of tibolone responsible for its estrogenic effects are widely distributed throughout the brain [39], many of these observed effects are due to the activation of these pathways, and this is highly dependent on the differential expression of oestrogen receptors in brain areas [119,120].

Finally, tibolone-induced transcriptional activation of neuroglobin is of clinical interest as a pharmacological therapy for AD. In a mouse model of AD, neuroglobin overexpression (Ngb-Tg x APP(Sw,Ind)) significantly reduced Aβ levels and improved animal behaviour in the Y-maze [121]. In support of the previous findings, Chen et al. (2012), using an animal model of Alzheimer’s disease (Tg2576), showed that neuroglobin overexpression reduced levels of hyperphosphorylated tau (amino acids T231 and S369), with this mechanism dependent on AKT by reducing GSK3B activation [122]. Neuroglobin therapy has many limitations, mainly due to its hydrophilic exterior and inability to cross the blood–brain barrier. To circumvent these clinical limitations, Li et al. (2015) injected transgenic AD mice (APPswe/PSEN1dE9) with a plasmid containing neuroglobin (pNgb, 1 mg/mL) by ICV once a week for 8 weeks and showed a significant improvement in memory and cognition when animals were tested in the Morris water maze [123], accompanied by a reduction in amyloid burden in both the cortex and hippocampus compared to animals injected with the vector alone (pCDNA3.1). From a translational perspective, neuroglobin at the extracellular level has been associated with amyloid plaques in CA1 of the hippocampus in early AD patients [124]. Surprisingly, neuroglobin levels change dramatically depending on the stage of pathology, being upregulated in early and moderate stages and significantly reduced in more severe stages of AD [124]. This evidence suggests that neuroglobin plays a critical regulatory role in disease progression and may influence the outcome, particularly in the later stages of the disease. Due to its limited ability to reach the brain, and considering that one of the protective functions attributed to tibolone is the regulation of neuroglobin, this compound may have enormous potential for repurposing in AD due to its broad effects mediated through oestrogen receptors, particularly β, without excluding the involvement of androgen and progesterone receptors.

## 5. Conclusions and Future Perspectives

**Figure 1:**
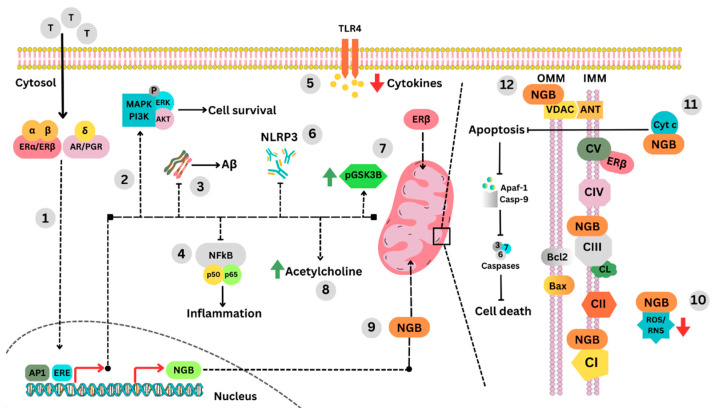
Neuroprotective actions of tibolone. Tibolone has neuroprotective mechanisms that act through its metabolism and interaction with various receptors. Once internalised, tibolone is metabolised by enzymes in the cytosol. The levels and activity of these enzymes may vary between cell types. The metabolites of tibolone, α, β, and Δ, exert estrogenic, androgenic, and progestogenic activity by binding to ERα, ERβ, AR, and PGR receptors in a tissue-dependent manner (1). Receptor-specific activation promotes the transcriptional activity of neuroprotective genes that stimulate MAPK/AKT-dependent pathways to promote cell survival (2). This can reduce Aβ accumulation (3), NF-kB nuclear translocation, (4) and Toll-like receptor 4 (TL4)-induced pro-inflammatory cytokines (5), leading to inhibition of NLRP3 inflammasome formation (6). Tibolone also prevents tau hyperphosphorylation by stimulating GSK3B phosphorylation (7) and has been shown to increase acetylcholine levels (8). This may improve short- and long-term memory processes. In addition, the estrogenic metabolites of tibolone activate ERβ, leading to the activation of a non-genomic mechanism directly on the mitochondria. ERβ has a mitochondrial localisation sequence that facilitates its translocation to this organelle. Once internalised, ERβ interacts with ATP synthase (CV), thereby enhancing metabolism and oxidative phosphorylation. Transcriptional regulation of neuroglobin (Ngb) is mediated in part by oestrogen receptors, mainly ERβ (9). Under challenging conditions, Ngb is translocated to different cellular compartments, such as the Golgi apparatus and the endoplasmic reticulum. However, the mitochondria are the main destination of this protein. At the mitochondrial level, Ngb plays a critical role by reducing oxidative mechanisms, neutralising ROS/NOS (10), inhibiting apoptosis activation by preventing cytochrome c translocation to the cytosol (11), and increasing Bcl2 (anti-apoptosis) while decreasing Bax (pro-apoptosis). Ngb also helps regulate the opening of the mitochondrial transition pore by physically interacting with VDAC (12). This prevents the loss of mitochondrial dynamics and integrity under the conditions of oxidative stress commonly seen in Alzheimer’s disease. Abbreviations: CI, complex I; CII, complex II; CIII, complex III; CIV, complex IV; CV, complex V or ATP synthase. VDAC, voltage-dependent anion channel; Ngb, neuroglobin; Cyt c, cytochrome c; ROS, reactive oxygen species; RNS, reactive nitrogen species; AP1, activating protein-1; ERE, oestrogen response elements; ERα, oestrogen receptor alpha; ERβ, oestrogen receptor beta; AR, androgen receptor; OGR, progesterone receptor; NLRP3, nucleotide-binding domain, leucine-rich–containing family, pyrin domain–containing-3; CL, cardiolipin.

As AD is a multifactorial disease, it is a challenge to research and implement specific treatments that can reduce the risk or slow the progression and development of the disease. In this context, tibolone may be of potential clinical benefit due to its vast anti-inflammatory, anti-apoptotic, and antioxidant effects, in addition to reducing Aβ and tau hyperphosphorylation by increasing levels of phosphorylated GSK3B (Figure 1).

Given the ineffectiveness of neuroglobin as a drug therapy, several alternatives have been proposed for its cerebral delivery, many of which encapsulate the protein, with all the limitations this entails (different methods, formulations, and denaturation of the protein); a better alternative would be to stimulate the endogenous regulation of its levels using a compound used to treat other diseases or pathological conditions, such as tibolone.

One limitation of tibolone as a therapy would be the so-called “therapeutic window” of its protective effects. Previous studies from the Women’s Health Initiative (WHI) have shown that current hormone therapy with oestradiol may worsen symptoms in AD patients compared with women treated soon after the diagnosis of menopause; it is possible that treatment with tibolone may have the same adverse effect. Another unanswered question is which metabolite of tibolone, α, β, or Δ, is responsible for neuroglobin induction and how long these effects last without interruption over time. Although the therapeutic window for tibolone to increase neuroglobin is a critical factor that may limit its therapeutic use, alternatives such as protein encapsulation for efficient and successful delivery have been implemented.

As explained above, tibolone exerts most of its anti-apoptotic and anti-inflammatory effects through steroid receptors, which are highly neuroprotective in several brain pathologies. The flexibility of this compound to activate specific classes of receptors and possibly different cell types and tissues in which it acts makes it difficult to study its mechanisms of action and the molecular pathways underlying its effects. These limitations should not be seen as an obstacle, but rather as a motivation to further investigate its utility and potential applicability in a disease as devastating as Alzheimer’s.

## Data Availability

Not applicable.
